# Airway and ventilation management during cardiopulmonary resuscitation and after successful resuscitation

**DOI:** 10.1186/s13054-018-2121-y

**Published:** 2018-08-15

**Authors:** Christopher Newell, Scott Grier, Jasmeet Soar

**Affiliations:** Intensive Care Unit, Southmead Hospital, North Bristol NHS Trust, Bristol, BS10 5NB UK

**Keywords:** Airway, Cardiac arrest, Cardiopulmonary resuscitation, Oxygenation, Ventilation

## Abstract

After cardiac arrest a combination of basic and advanced airway and ventilation techniques are used during cardiopulmonary resuscitation (CPR) and after a return of spontaneous circulation (ROSC). The optimal combination of airway techniques, oxygenation and ventilation is uncertain. Current guidelines are based predominantly on evidence from observational studies and expert consensus; recent and ongoing randomised controlled trials should provide further information. This narrative review describes the current evidence, including the relative roles of basic and advanced (supraglottic airways and tracheal intubation) airways, oxygenation and ventilation targets during CPR and after ROSC in adults. Current evidence supports a stepwise approach to airway management based on patient factors, rescuer skills and the stage of resuscitation. During CPR, rescuers should provide the maximum feasible inspired oxygen and use waveform capnography once an advanced airway is in place. After ROSC, rescuers should titrate inspired oxygen and ventilation to achieve normal oxygen and carbon dioxide targets.

## Background

Airway and ventilation interventions during cardiopulmonary resuscitation (CPR) and in those with a return of a spontaneous circulation (ROSC) follow a stepwise approach as the precise interventions are thought to depend on patient factors, rescuer skills and the stage of the resuscitation [1, 2]. Current guidelines for in-hospital cardiac arrest (IHCA) and out-of-hospital cardiac arrest (OHCA) are based primarily on evidence from observational studies and expert consensus, and the optimal interventions remain uncertain [3–5]. In addition, our knowledge of airway management during IHCA is mainly extrapolated from OHCA studies.

## Do we need an airway, oxygenation and ventilation during CPR?

Current guidelines recommend that, after a primary cardiac arrest, restoring a circulation with chest compressions and, if appropriate, attempted defibrillation to restart the heart take priority over airway and ventilation interventions [2, 4]. The premise is that there is an adequate oxygen reservoir at the time of cardiac arrest and further oxygen is only required after about 4 minutes. When cardiac arrest follows airway and/or breathing problems (asphyxial cardiac arrest), earlier interventions to restore adequate oxygenation to the vital organs may be preferable.

Current guidelines for CPR [2–4, 6] emphasise chest compressions for all cardiac arrests because:

- Chest compressions are easy to learn and do for most rescuers and do not require special equipment. Studies show that lay rescuer compression-only CPR is better than no CPR [7].
- Sudden cardiac arrest, with an initial shockable rhythm (ventricular fibrillation or pulseless ventricular tachycardia [VF/pVT]) has good outcomes with early CPR and early defibrillation [8].
- Survival after a non-cardiac cause of cardiac arrest, such as asphyxial cardiac arrest and which more commonly lead to an initial non-shockable cardiac arrest rhythm (pulseless electrical activity (PEA) or asystole), is relatively poor even if there is ROSC. Patients often have severe brain injury associated with hypoxaemia and low blood flow preceding cardiac arrest, a period of no or low flow during CPR and reperfusion injury following ROSC.
- As VF/pVT has a better response to treatment, CPR interventions prioritise treatment for VF/pVT at the expense of those that may be helpful for PEA or asystole.

Observational data suggest that early lay-bystander compression-only CPR can improve survival after sudden cardiac arrest [9]. This could be because of an increased likelihood of bystanders performing compression-only CPR rather than no CPR, or CPR with long pauses for probably ineffective ventilation attempts. In addition, dispatch-assisted compression-only CPR appears to give similar or improved outcomes compared with dispatcher CPR instructions for both compressions and ventilations [5]. Additional benefits of CPR with compressions and ventilations are most likely when delivered by rescuers trained in ventilation, when emergency medical service (EMS) response times are long or after an asphyxial cardiac arrest [2, 6].

Some EMS services deliver continuous high-quality chest compressions with passive oxygenation with an oropharyngeal airway and simple oxygen mask (minimally interrupted cardiac resuscitation) and an advanced airway is delayed until after 600 chest compressions for witnessed OHCA with a shockable rhythm. Observational studies show improved survival to discharge for all adult OHCAs, and improved survival with good neurological outcome for witnessed cardiac arrest or if the initial rhythm is shockable [10]. Whether chest compressions generate a sufficient tidal volume for gas exchange is uncertain and likely to vary over time. Studies in late cardiac arrest (40–50 minutes) show that the tidal volumes generated are less than the patient’s estimated deadspace [11].

## Steps for airway and ventilation management during CPR and after ROSC

**Fig. 1:**
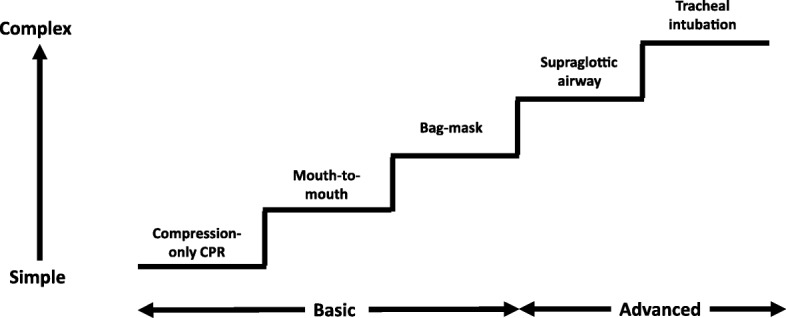
Stepwise approach to airway management during cardiopulmonary resuscitation

During CPR, airway interventions range from compression-only CPR with or without airway opening, mouth-to-mouth ventilation, mouth-to-mask ventilation, bag-mask ventilation (with or without an oropharyngeal airway) or advanced airways (supraglottic airways (SGAs) and tracheal intubation using direct or video laryngoscopy) (Fig. 1). In a feasibility study to inform a randomised controlled trial (RCT) of OHCA, patients in the ‘usual’ airway management group were observed to have both basic and advanced airway interventions which changed according to the skills of the rescuer present and the time-point during resuscitation [12].

After ROSC for both IHCA and OHCA, most patients have a post-cardiac arrest syndrome [13], are comatose with impaired airway reflexes and ventilation and/or have an indication for tracheal intubation based on their underlying condition [14, 15]. Patients who remain conscious and do not require airway interventions tend to have an initial shockable rhythm, are treated early with defibrillation and have better outcomes. Tracheal intubation enables controlled ventilation to facilitate onwards transportation to the emergency department after OHCA, cardiac catheterisation laboratory or intensive care unit. Drug-assisted intubation by critical care teams for both IHCA and OHCA patients with ROSC using a protocol-based approach (e.g. with ketamine or midazolam, fentanyl and rocuronium) can be safe and effective [16, 17].

## Airway and ventilation techniques during CPR

### Bag-mask ventilation

On arrival of trained rescuers, bag-mask ventilation with supplemental oxygen is the most common initial approach and can be aided with an oropharyngeal or nasopharyngeal airway. During CPR, the bag-mask is used to give two breaths after every 30 compressions. A large RCT of bag-mask ventilation without pausing compressions in OHCA found no difference in survival when compared with pausing for ventilation after every 30 compressions [18]. A pre-specified per-protocol analysis reported a significantly higher survival to discharge among those who actually received conventional CPR (30:2) compared with those who received continuous compressions.

### Supraglottic airways

Supraglottic airway (SGA) use has increased during CPR as SGA insertion is easier to learn than tracheal intubation and feasible with fewer and shorter interruptions in chest compression [19]. Observational data show classic laryngeal airway mask (cLMA) use during CPR is associated with a lower incidence of regurgitation of gastric contents than bag-mask ventilation [20]. Second-generation SGAs (e.g. i-gel and LMA Supreme (LMAS)) have potential advantages over first-generation SGAs, including improved pharyngeal seal pressure, oesophageal drainage tubes and integrated bite blocks. A pig study raised concerns that a supraglottic cuff compresses the internal and external carotid artery, decreasing cerebral blood flow during CPR. A human radiographic study did not, however, observe any evidence of mechanical compression of the carotid arteries [21, 22].

### Tracheal intubation

Tracheal intubation enables chest compressions to continue uninterrupted while the lungs are ventilated, avoids gastric insufflation and protects the lungs from aspiration of gastric contents: an observational study, however, showed one-third of OHCA patients had regurgitation, and in two-thirds this occurred before EMS arrival and in a quarter between EMS arrival and tracheal intubation [23]. Studies suggest more than 50 successful intubations are required to achieve an insertion success rates of over 90% during CPR [24]. Current European guidelines recommend a pause in compressions of less than 5 s for tracheal tube insertion [1].

Videolaryngoscopy (VL) for tracheal intubation may have a role in tracheal intubation during CPR [25], although there are few studies of VL use during CPR. In one study of experienced clinicians, VL was associated with significantly fewer episodes of prolonged (> 10 s) interruptions in chest compressions; the intubation success rate was not significantly different [26]. In a further study, VL use was associated with shorter pauses in compressions compared with direct laryngoscopy when initial tracheal intubation was not successful [27].

## Comparisons between airway techniques during CPR

Comparisons between airway techniques are difficult as most patients have more than one airway technique during CPR [12], airway interventions depend on patient and event factors that are not reported (e.g. arrest location and access, obesity), rescuer ability determines technique success and early-ROSC patients are less likely to need an advanced airway.

### Basic versus advanced airways during CPR

Available evidence challenges the notion that ‘advanced’ (SGA or tracheal tube) interventions are better than ‘basic’ (bag-mask ventilation) interventions during CPR. Meta-analysis of observational studies of OHCA estimated an advanced airway was associated with a reduced survival to hospital discharge/30 days (odds ratio 0.49 (95% confidence interval (CI) 0.37–0.65)) when compared with bag-mask ventilation [28]. Observational studies are likely to be confounded because, if ROSC occurs early, an advanced airway during CPR may not be required whereas patients with primary asphyxial cardiac arrest or aspiration of gastric contents tend to get an advanced airway and have a poorer outcome.

The Cardiac Arrest Airway Management (CAAM) multi-centre RCT randomised 2043 OHCA patients to early tracheal intubation or bag-mask ventilation with delayed post-ROSC tracheal intubation, delivered by a physician-led prehospital care team [29]. Bag-mask compared to tracheal tube use failed to show non-inferiority or inferiority for favourable 28-day survival with neurological function (4.3 versus 4.2%). The authors report this as an ‘inconclusive result’. The bag-mask group had more airway complications: difficult airway management (18.1 vs 13.4%, *P* = 0.004), failure (6.7 vs 2.1%, *P* < 0.001) and regurgitation of gastric contents (15.2 vs 7.5%, *P* < 0.001). Oesophageal intubation was recognised and corrected in 10.2% of cases.

No large RCTs of airway management for IHCA have been conducted. Time-dependent propensity analysis of data from the American Heart Association Get With The Guidelines IHCA registry showed tracheal intubation during each of the first 15 minutes of resuscitation compared with no intubation during that minute was associated with decreased survival to hospital discharge [30]. This study using observational data could not correct for a number of confounders (e.g. skills and experience of rescuers, the cause of the cardiac arrest, CPR quality and the indication for intubation) and confounding by indication could influence the results. This study raises the possibility that early tracheal intubation could be harmful during CPR after IHCA and highlights the need for RCTs of IHCA airway management.

### Supraglottic airways versus tracheal intubation during CPR

A meta-analysis of ten observational studies with 76,000 patients reported an association between tracheal intubation and an increased rate of neurologically intact survival (OR 1.33, CI 1.09–1.61) compared with SGA use [31]. A feasibility study of 615 OHCA patients to help inform a larger RCT randomised paramedics to use an i-gel, LMAS or usual care (most commonly tracheal intubation) [32]. This feasibility study, which is one of the largest RCTs of advanced airway management during CPR, found no difference in survival to discharge (i-gel 9.5%, LMA supreme 6.9%, usual care 8.6%) or 90 days (9.5% vs 6.9%), neurocognitive function or quality of life between groups, but was not powered to detect clinically significant differences in these outcomes. Recruitment to the LMAS group was stopped because on three occasions rescuers were contaminated as chest compressions caused blood and vomit to be ejected forcefully from the LMAS gastric drainage port. First attempt placement success rates were 79% for the i-gel and 75% for the LMAS, and the first attempt tracheal intubation rate was 85%. In an observational study of OHCA, successful placement of the laryngeal tube occurred in 85% of 344 patients [33].

A commonly cited reason against using a tracheal tube during CPR is that insertion leads to prolonged and potentially harmful interruptions in chest compression. In an observational study of 100 pre-hospital intubations by paramedics, tracheal intubation attempts during CPR caused a median 110 s (IQR 54–198 s) of interruption, and in a quarter of cases the interruptions were over 3 minutes [19]. More recent OHCA observational data (339 patients) suggest duration of the longest pauses, number of pauses over 10 s and chest compression fraction (proportion of time compressions being given) may be similar with bag-mask, SGA and tracheal intubation [27]. In addition, data from 2767 cases of OHCA suggest the chest compression fraction is only slightly less with a tracheal tube (72.4 vs 76.7%) [34].

Finally, the Pragmatic Airway Resuscitation Trial (PART) cluster randomised trial comparing tracheal intubation with laryngeal tube (LT) insertion in 3005 OHCA patients has reported its initial results (presented at the Society for Academic Emergency Medicine, 16 May 2018) [35]. EMS were randomised in clusters with crossover at 3–5-month intervals. The primary end-point of 72-h survival was improved with LT compared with tracheal intubation (18.2 vs 15.3%, adjusted difference 2.9% (95% CI 0.2–5.6%), *P* < 0.01), as were the secondary outcomes of ROSC (27.9 vs 24.1%, *P* = 0.02), hospital survival (10.8 vs 8.0%, *P* = 0.01) and favourable neurological status at discharge (7.0 vs 5.0%, *P* = 0.02). The full results of PART and another large RCT that compares i-gel with tracheal intubation during OHCA are awaited [36].

## The role of waveform capnography during CPR and after ROSC

Waveform capnography is recommended whenever an advanced airway (SGA or tracheal tube) is used both during CPR and after ROSC [1]. A SGA will provide reliable end-tidal carbon dioxide values (ETCO_2_) when there is a good seal. The ETCO_2_ depends on a large number of physiological variables (including cardiac output, metabolic state, lung function). This will lead to some limitations in the usefulness of ETCO_2_ monitoring during CPR and after ROSC. Waveform capnography has the following roles during CPR:

1. Confirms correct tracheal tube placement [4].
2. Helps guide rescuers to ventilate at the correct rate, although chest compression artefacts may lead to a falsely high ventilation rate [37].
3. Helps guide chest compression quality. A recent study suggests an association between high-quality chest compressions with a higher ETCO_2_ and defibrillation success after OHCA [38].
4. Helps identify ROSC during CPR. An increase in ETCO_2_ during CPR or a rising trend may indicate ROSC [39].
5. Helps make decisions about stopping CPR. A systematic review of 17 observational studies observed an association between a low ETCO_2_ (< 10 mmHg at 20 minutes) with a low likelihood of ROSC (< 0.5%) [40]. Given the large number of factors that can influence the ETCO_2_, trends in ETCO_2_ during CPR rather than single values may be more important to guide decisions. In addition, a multi-modal approach rather than the ETCO_2_ alone should be used in prognostic decisions during CPR.

Waveform capnography helps guide ventilation rate and correct tracheal tube placement after ROSC. Post-ROSC patients often have a poor cardiac output and a large alveolar deadspace and this effects the correlation between ETCO_2_ and arterial partial pressure of carbon oxygen (PaCO_2_). In an arterial blood gas study, the median (interquartile range) PaCO_2_ was 67 (34) mmHg and ETCO_2_ 31 (25) mmHg during CPR, and after ROSC the PaCO_2_ was 58 (21) mmHg and ETCO_2_ 37.5 (17) mmHg [41]. Data from the TTM study show patients managed at 33 °C have a lower ETCO_2_ than those at 36 °C. Observational studies show an increased PaCO_2_ to ETCO_2_ gap both during CPR and after ROSC is associated with decreased ROSC and survival to hospital discharge, respectively [41, 42].

## How much oxygen during CPR and after ROSC?

The optimal oxygen requirement for CPR and after ROSC remains uncertain [43]—too little is harmful, too much could be harmful, and what’s just right and how it should be measured and targeted are uncertain.

Current guidelines recommend giving the maximum feasible inspired oxygen during CPR based on the premise that restoring depleted oxygen levels and correcting tissue hypoxia improves survival. Observational data show an association between higher arterial oxygen partial pressures during CPR and improved ROSC [1, 4, 41, 44]. Due to the low flow cardiac output state, despite administration of a high inspired fraction of oxygen, target tissue mitochondrial oxygen tension is unlikely to be high [45].

After ROSC, the inspired oxygen should be titrated to achieve normal oxygen saturations (94–98%) once oxygenation can be reliably monitored with pulse oximetry [4, 46]. Observational studies show that hypoxia after ROSC is associated with a decrease in survival to hospital discharge [47–49]. The effect of hyperoxia after ROSC is less certain. Post-cardiac arrest syndrome includes reperfusion injury and oxidative stress, which can lead to neuronal damage. Hyperoxia is thought to further increase oxidative stress [45]. Animal studies show that hyperoxia immediately after ROSC is associated with a worse neurological outcome [50]. A small RCT of 28 OHCA patients showed a greater rise in neuron-specific enolase (NSE), a serum marker for neuronal injury, in post-ROSC patients treated with 100% inspired oxygen compared with 30% inspired oxygen for 60 minutes after ROSC (neither group received any temperature control) [51]. Several studies show an association between hyperoxia and worse outcome at hospital discharge (overall survival, or survival with good neurological function) when compared with normoxia, while others report no association [4, 47, 49, 52–57]. These studies are difficult to interpret as a high inspired oxygen may be a surrogate marker of illness severity, the studies have not looked at oxygenation immediately after ROSC (the time period where animal studies show harm), the actual duration (‘dose’) of hyperoxia for an individual patient is unknown and the impact of other interventions (e.g. temperature control, carbon dioxide target) is uncertain. A feasibility study of titrated oxygen immediately after ROSC struggled to reliably measure oxygen saturation to enable titration of inspired oxygen using a bag-mask [58]. An RCT of titrating oxygen immediately after ROSC is about to start (Table 1).

**Table 1:** Randomised controlled trials in progress

Title	Country	Summary	Current status	Registration number	Links
AIRWAYS-2 randomised controlled trial of i-gel supraglottic airway device versus tracheal intubation in the initial airway management of out of hospital cardiac arrest [36]	United Kingdom	Cluster randomised trial comparing tracheal intubation with supraglottic airway (i-gel) insertion as the initial airway in out-of-hospital cardiac arrest. Primary outcome: neurological outcome at hospital discharge or at 30 days if still hospitalised	Enrolment completed August 2017	ISRCTN08256118	https://www.ncbi.nlm.nih.gov/pubmed/27697605
Pragmatic Airway Resuscitation Trial (PART) [35]	United States of America	Cluster randomised trial comparing tracheal intubation and laryngeal tube insertion in out-of-hospital cardiac arrest. Primary outcome: 72-h survival	Enrolment completed 2017	NCT02419573	https://www.ncbi.nlm.nih.gov/pubmed/26851059 Initial results presented and available at https://www.eventscribe.com/2018/SAEM/agenda.asp?h=Plenary&BCFO=PL (accessed 31 May 2018)
Reduction of oxygen after cardiac arrest: The EXACT trial	Australia	Phase 3 Randomised controlled trial comparing oxygen titrated to saturations of 90–94% with 98–100% as soon as possible after ROSC and continued until ICU admission. Primary outcome: survival to hospital discharge.	Enrolment starts October 2017	NCT03138005	https://clinicaltrials.gov/ct2/show/record/NCT03138005?term=Reduction+of+oxygen+after+cardiac+arrest&rank=1
Targeted therapeutic mild hypercapnia after resuscitated cardiac arrest (TAME)	Australia	Randomised controlled trial comparing mild hypercapnia (PaCO_2_ 50–55 mmHg) with targeted normocapnia (PaCO_2_35–45 mmHg). Primary outcome: neurological outcome at 6 months (GOSE)	Enrolment starts December 2017	NCT03114033	https://clinicaltrials.gov/ct2/show/NCT03114033
Targeting low- or high-normal Carbon dioxide, Oxygen, and Mean arterial pressure After Cardiac Arrest and REsuscitation: study protocol for a randomised pilot trial [74]	Finland	Feasibility study	Enrolment complete	NCT02698917	https://clinicaltrials.gov/ct2/show/NCT02698917

## How much ventilation during CPR and after ROSC?

In the absence of an advanced airway during CPR, current guidelines based on very limited evidence recommend two positive pressure breaths after every 30 chest compressions. These breaths should be of an inspiratory time of 1 s and produce a visible chest wall rise [59]. Observations in anesthetised adults show a visible chest rise occurs with a mean tidal volume of 384 ml (95% CI 362 to 406 ml) [60]. Once an advanced airway is in place, a ventilation rate of 10 min^− 1^ without interrupting chest compressions is recommended. Continuous uninterrupted chest compressions are not always feasible with a SGA and there may be a need to pause after every 30 chest compressions in order to give two rescue breaths.

Our understanding of the optimal ventilation strategy and its interaction with chest compressions to generate adequate blood flow and oxygen delivery to vital organs is limited [61]. The recommended ventilation rate of 10 min^− 1^ with a tracheal tube is based predominantly on animal studies, which followed observations that hyperventilation was common during human CPR [62]. A pig study showed a respiratory rate of 30 min^− 1^ compared to 12 min^− 1^ caused increased intrathoracic pressure, a decrease in coronary and cerebral perfusion and decreased ROSC [63, 64]. Furthermore, the authors included human observational data and reported no survivors from cardiac arrest with an advanced airway when the respiratory rate was greater than 10 min^− 1^ and the inspiratory time greater than 1 s. A reduced ventilation rate may be sufficient to maintain a normal ventilation perfusion ratio during CPR as the cardiac output generated by chest compressions is also markedly reduced.

The interaction between the lungs and circulation during CPR are complex [61]. Increasing ventilation rate or tidal volume during CPR increases the mean intrathoracic pressure and reduces venous return to the heart, increases lung volume and pulmonary vascular resistance, reduces cardiac output and decreases coronary perfusion pressure and aortic blood pressure. Devices designed to regulate intrathoracic pressure such as the impedance threshold device (ITD) and active compression decompression CPR devices (ACD CPR) aim to augment blood flow to the heart and brain during CPR. Specifically, the ITD stops airflow into the lungs during chest compression recoil or active decompression and the negative resultant intrathoracic pressure increases blood flow into the ventricles. Compared with standard CPR, ITD CPR and ACD + ITD CPR augment cardiac output for the next compression [61]. Despite the promising effects of ITD + ACD CPR in animal models, the results from human trials are less convincing. Studies of the ITD alone show no improvement in survival. The International Liaison Committee on Resuscitation (ILCOR) 2015 review of the science of ACD + ITD CPR did not achieve consensus regarding its use, albeit a large RCT had reported improved survival with good neurological function [4].

Current guidelines for post-ROSC care recommend using low tidal volume ventilation (6–8 ml kg^− 1^ IBW) with titrated levels of PEEP and aiming for normocapnia [46]. After ROSC, inadequate ventilation and resultant hypercapnia will exacerbate any existing metabolic acidosis and potentially worsen any haemodynamic instability. In addition, hypercapnia produces cerebral vasodilatation if cerebrovascular reactivity is preserved: whether this is detrimental or beneficial is not known. Hypercapnia may lead to an elevation in intracranial pressure and worsening of hyperaemia in a vulnerable brain, or increased blood flow may improve cerebral ischaemia and be neuroprotective. One observational study showed improved survival to hospital discharge and neurological outcomes associated with exposure to mild hypercapnia compared to normocapnia or hypocapnia [65], whereas another showed worse survival to discharge with hypercapnia compared to normocapnia or hypocapnia [49]. In a small RCT of 86 post-cardiac arrest patients there was a greater increase in NSE (a marker of neuronal injury) in the first 72 h when normocapnia (35–45 mmHg, 4.67–6.0 kPa) was targeted compared to mild hypercapnia (50–55 mmHg, 6.67–7.33 kPa) [66]. This study is being followed up with a larger multi-centre RCT (The TAME Cardiac Arrest trial). Hyperventilation and hypocapnia may also cause cerebral ischaemia as a result of cerebral vasoconstriction, cerebrospinal fluid alkalosis and increased neuronal excitability due to increased excitatory amino acid release [67]. A ten-patient study showed decreased cerebral tissue oxygenation monitored by near infrared spectroscopy when the target PaCO_2_ decreased from 40 (5.33 kPa) to 30 mmHg (4.0 kPa) in post-ROSC patients treated with hypothermia [68]. A study of 5258 patients (82 ICUs in the Netherlands) observed a risk-adjusted increased mortality with hypocapnia compared with normocapnia and hypercapnia [69].

A post-ROSC lung protective ventilation strategy is based on guidance for acute lung injury ventilation. One study comparing a tidal volume less than or greater than 8 ml kg^− 1^ in OHCA survivors observed a lower tidal volume in the first 48 h post-ROSC was associated with a favourable neurocognitive outcome, more ventilator and shock-free days [70], whereas an IHCA study found no association between a tidal volume of less or greater than 8 ml kg^− 1^ in the first 6 or 48 h post-ROSC and survival to discharge and neurological outcome [71]. In the TTM trial, the end of TTM median tidal volume was 7.7 ml kg^− 1^ predicted body weight, 60% of patients had a tidal volume less than 8 ml kg^− 1^, median PEEP was 7.7 cmH_2_O (6.4–8.7), mean driving pressure was 14.6 cmH_2_O (± 4.3) and median FiO_2_ was 0.35 (0.30–0.45) [72]. Non-survivors compared with survivors at 28 days had worse oxygenation, higher respiratory rates, driving pressures and plateau pressures and lower compliance compared to survivors.

After ROSC, interventions for oxygenation and ventilation in combination with a bundle of interventions that adjust other physiological variables, including temperature, blood pressure, glucose and seizure control, are probably required for a good outcome [73]. The optimal targets and combinations are uncertain and the subject of ongoing studies [74].

## Randomised controlled trials in progress

There is clinical equipoise regarding the optimal airway, ventilation and oxygenation strategy during CPR and after ROSC. Several RCTs are currently in progress and these studies are summarised in Table 1.

## Conclusions

The optimal combination of airway techniques and oxygen and ventilation targets during CPR and after ROSC is uncertain. In the absence of evidence to favour a specific technique, rescuers should use the airway technique they are most proficient in during CPR and give the maximum feasible inspired oxygen concentration. Patients usually receive a stepwise approach as expert help arrives (Fig. 1). A compression to ventilation ratio of 30:2 should be used until an advanced airway is inserted, when a ventilation rate of 10 min^− 1^ should be used without interrupting chest compressions. After ROSC, oxygenation and ventilation should be titrated to achieve normal values. Ongoing RCTs (Table 1) should provide new insights.

## Abbreviations

ACD: Active compression-decompression
CO_2_: Carbon dioxide
CPR: Cardiopulmonary resuscitation
DL: Direct laryngoscopy
EMS: Emergency medical service
ETCO_2_: End-tidal carbon dioxide
FiO_2_: Fraction inspired oxygen
IBW: Ideal body weight
IHCA: In-hospital cardiac arrest
IQR: Inter-quartile range
ITD: Impedance threshold valve
NSE: Neuron specific enolase
OHCA: Out-of-hospital cardiac arrest
PaCO_2_: Partial pressure of arterial carbon dioxide
PEEP: Positive end expiratory pressure
RCT: Randomised controlled trial
ROSC: Return of spontaneous circulation
SGA: Supraglottic airway
TTM: Targeted temperature management
VF/pVT: Ventricular fibrillation/pulseless ventricular tachycardia
VL: Videolaryngoscopy
